# Ultra-Wideband Angle of Arrival Estimation Based on Angle-Dependent Antenna Transfer Function

**DOI:** 10.3390/s19204466

**Published:** 2019-10-15

**Authors:** Anton Ledergerber, Raffaello D’Andrea

**Affiliations:** Institute for Dynamic Systems and Control, ETH Zurich, 8092 Zurich, Switzerland

**Keywords:** angle of arrival, direction of arrival, ultra-wideband, channel impulse response, antenna transfer function, localization, machine learning, neural network

## Abstract

Ultra-wideband radio signals are used in communication, indoor localization and radar systems, due to the high data rates, the high resilience to fading and the fine temporal resolution that can be achieved with a large bandwidth. This paper introduces a new method to estimate the angle of arrival of ultra-wideband radio signals with which existing time-of-flight based localization and radar systems can be augmented at no additional hardware cost. The method does not require multiple transmitter or receiver antennas, or relative motion between transmitter and receiver. Instead, it is solely based on the angle-dependent impulse response function of ultra-wideband antennas. Datasets on which the method is evaluated are publicly available. The method is further applied to a localization problem and it is shown how a robot can self-localize solely based on these angle of arrival estimates, and how they can be combined with time-of-flight measurements. Even though existing angle of arrival techniques that use multiple antennas show better accuracy, the method presented herein looks promising enough to be developed further and could potentially lead to electronically and mechanically simpler angle of arrival estimation technology.

## 1. Introduction

Angle of arrival (AOA) measurement technologies for electromagnetic waves are employed in a wide range of applications. Both biomedical and military radar technology aim to measure not only the distance, but also the angle to objects reflecting electromagnetic waves. Wireless communication equipment, such as Wi-Fi routers, often estimate the angle of arrival to beamform their radiation to other network devices, and aircrafts use the AOA measurements to stationary beacons to localize themselves by means of automatic direction finders.

Traditionally, AOA measurement techniques require either a rotating directional antenna or multiple locations at which the signal is measured. These multiple measurement points are realized either by moving a single antenna to different locations while collecting signal measurements, or by multiple antennas, e.g., in the form of an antenna array. Often, these antenna arrays have a limited field from which they can estimate the AOA of a signal and therefore need to be additionally rotated. This leads to high performance, but also electronically and mechanically more complex and more costly devices, which has made AOA localization approaches less suitable for low-cost ultra-wideband (UWB) localization systems [1].

The AOA measurement technique presented in this paper proposes to make use of the angle-dependent antenna transfer function, which manifests itself in the measured channel impulse response (CIR). Doing so, it enables estimation of the angle of arrival using a single, static antenna, requiring no additional hardware. Compared to our preliminary work [2] we provide more detailed analytical and experimental results, showing

- that the AOA estimation method is not bound to a specific environment,
- that the method also works without reflective surfaces in the receiver antenna’s vicinity if the antenna is chosen accordingly, and
- how the proposed method can be integrated with existing time of flight (TOF)-based localization methods, with which training data can be acquired on the go.

Furthermore, the data from the experiments presented in this paper are made available [3], such that other estimation strategies could be tested on them.

### 1.1. Outline

This paper is structured as follows: In the remainder of this section, we review related work. Section 2 discusses how the antenna transfer function and the environment influence the measured CIR between a transmitter and a receiver. Based on these insights, a machine learning approach mapping a measured CIR to an AOA probability distribution is presented in Section 3. The data on which the method is tested is discussed in Section 4 and results are shown in Section 5. The method is then applied to a localization problem in Section 6. Concluding remarks are made in Section 7. Note that throughout the paper a two-dimensional problem setup is considered, if not stated otherwise.

### 1.2. Related Work

An overview of AOA estimation methods is given in [4]. Common methods measure the phase difference of arrival of a signal with two or more antennas integrated into an antenna array (as for example done in [5]) or employ beamforming techniques to steer the main radiation lobe(s) of the antenna array towards the angle of arrival [6]. Algorithms fusing the outputs of such multi-port antenna arrays are discussed in [7,8]. Aside from conventional algorithms, recently deep learning has also been applied to process the output of antenna arrays for estimating the AOA. Its advantages compared to traditional methods when estimating multiple signal sources and their AOA are discussed in [9]. Beamforming control by means of deep learning is discussed in [10] where it is also shown how neural network quantization facilitates the deployment of such deep learning techniques in low-memory, low-overhead platforms such as mobile phones.

Modern Wi-Fi modules are often equipped with two or more antennas and provide the phase shifts of the different sensing elements in the channel state information (CSI). Impressive localization results based on such CSI measurements were obtained in [11], where it is shown how localization can be achieved via multipath triangulation and time-of-flight-difference (TOFD) measurements using a single Wi-Fi module employing three antennas.

Alternatives to multi-port antenna arrays for beam steering and AOA estimation include single-port switched parasitic antenna arrays (as for example discussed in [12]) or rotating directional antennas or antenna arrays, as employed by classical radars where each direction is scanned for an incoming signal [13].

Instead of employing rotating antennas or antenna arrays, it is also possible to estimate the AOA by collecting signals from the same source sequentially at multiple locations or during movements similar to the synthetic aperture radar. This principle is utilized in [14] to estimate the AOA using received signal strength measurements, and in [15] using phase measurements, collected at different locations.

Compared to the previously discussed approaches to estimate the AOA, the method proposed herein only relies on CIR measurements acquired by a single static antenna at a single location using no actively controlled parasitic elements. It is based on the angle-dependent antenna transfer function, which leaves its mark in the measured CIR of a UWB propagation channel. UWB propagation channels in general are discussed in [16], and in [17,18,19] with a focus on the antenna transfer functions. Distortions of the measured CIR due to angle-dependent antenna transfer functions can lead to angle-dependent errors in the timestamps provided by leading edge detection algorithms. In turn, these angle-dependent errors in the timestamps lead to errors in the TOF or TOFD measurements. So far research in UWB localization has therefore focused either on tailored UWB antenna design [20,21] or on mitigating these effects via models predicting the systematic error. A neural network predicting the error in the TOF measurement based on CIR measurements was employed to this end in [22] while in the authors’ previous work [23] this error is predicted based on the AOA. Instead of compensating for effects of the angle-dependent transfer functions, this paper proposes to amplify them such that they can be exploited to estimate a signal’s AOA.

## 2. Channel Impulse Response

### 2.1. Components of the CIR

**Figure 1:**
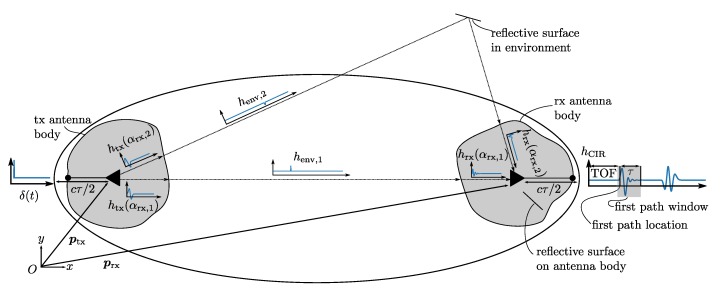
Composition of an example channel impulse response (CIR) for a propagation channel with one direct and indirect path and an angle of arrival (AOA)-dependent antenna impulse response function as given in Equation (1). By considering only a window of the CIR around the first path location, multipath components resulting from reflections outside the ellipsoid as given in Equation (3) are discarded.

The CIR, which fully characterizes a UWB propagation and communication channel, is subject to many influences: the transmitting antenna’s impulse response function $h_{\mathrm{tx}}$, the impulse response of the environment $h_{\mathrm{env}}$, and the receiving antenna’s impulse response function $h_{\mathrm{rx}}$, not to mention the influence of all the electronics involved in reading and writing to the antenna. By assuming a cascaded, linear, time invariant model [24], and a UWB channel with $N_{\mathrm{MP}}$ different multipath components [16], the channel impulse response is given as

(1) $$\begin{array}{c} h_{\mathrm{CIR}}=\sum_{n=1}^{N_{\mathrm{MP}}}h_{\mathrm{tx}}\left(\alpha_{\mathrm{tx},n}\right)*h_{\mathrm{env},n}*h_{\mathrm{rx}}\left(\alpha_{\mathrm{rx},n}\right), \end{array}$$

where the dependence of the antenna’s impulse response on the angle of departure (AOD) $\alpha_{\mathrm{tx},n}$ and the AOA $\alpha_{\mathrm{rx},n}$ of the *n*-th multipath component are explicitly written, and where * is the convolution operator. This is illustrated in Figure 1. We divide objects into two groups, objects belonging to the same rigid body as the transmitting or receiving antenna, and other objects, which are typically further away from the antenna as visualized in Figure 1. In the following, we absorb the influence of the first group of objects into the impulse response of the transmitting or receiving antenna as their influence is also angle-dependent.

While a UWB communication channel is difficult to model accurately due to the many influences [16] that need to be considered, it is possible to measure its CIR quite accurately by exciting the system with a pseudo noise sequence [25]. This principle is also employed by the DW1000 UWB chip [26], which will be used in the following to investigate the influence of the AOA on the complex CIR envelope it measures. The complex CIR envelope $h_{\mathrm{CIR}}$ is related to the actual real-valued CIR $h_{\mathrm{CIR}}$ by

(2) hCIR(t)=RehCIR(t)ej2πf0t,

with Re(·) denoting the real part, and with $f_{0}$ denoting the carrier frequency of the UWB signal as explained in more detail in [25] (p. 281).

### 2.2. Measuring the CIR for Different AOA

**Figure 2:**
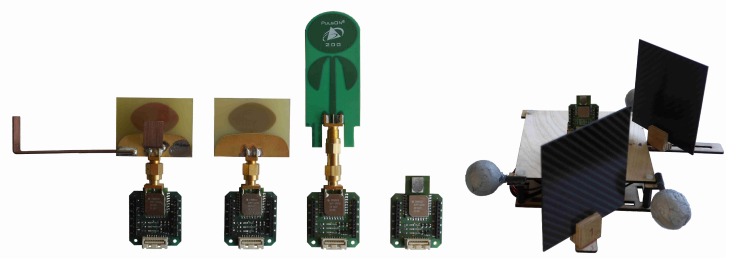
From left to right: A DWM1000 module equipped with a modified spline antenna, a spline antenna, a Broadspec Time Domain antenna, a Partron dielectric chip antenna. On the extreme right is a Partron dielectric chip antenna with carbon plates attached in its vicinity and the motion capture markers. This setup without the carbon plates is also used to collect measurements with the other antennas.

As explicitly indicated in Equation (1), the measured CIR is generally dependent on the AOA and AOD. In order to qualitatively assess this dependency (no anechoic chamber was used), CIR envelope measurements were gathered in a static environment with two UWB modules. A first UWB module had a fixed position and orientation, hereafter referred to as the anchor, whereas a second module, hereafter referred to as the tag, was rotated around its bore while it collected CIR measurements and range measurements to the anchor. At the same time, an overhead motion capture system recorded the position and orientation of both antennas. The ranging protocol presented in [27] was used and the CIR envelope obtained from the last anchor reply was recorded on the tag. This was done for five different tag antenna configurations, namely a Broadspec Time Domain Antenna [28], a spline antenna analyzed in detail in in [18], this spline antenna with copper arms soldered to its ground, and a Partron Dielectric Chip Antenna [29] with and without carbon plates in its vicinity, all shown in Figure 2. The Broadspec Time Domain antenna was used on the transmitter. All these measurements were made using DWM1000 modules [29], which were modified to allow connection of antennas other than the Partron dielectric chip antenna with which they are shipped. The DW1000 chip estimates the complex CIR envelope with a resolution of $T_{s}=1/\left(2f_{c}\right)\approx 1ns$, where $f_{c}=499.2\mathrm{MHz}$ is the chipping frequency [30]. Its configuration is discussed in Appendix A.

**Figure 3:**
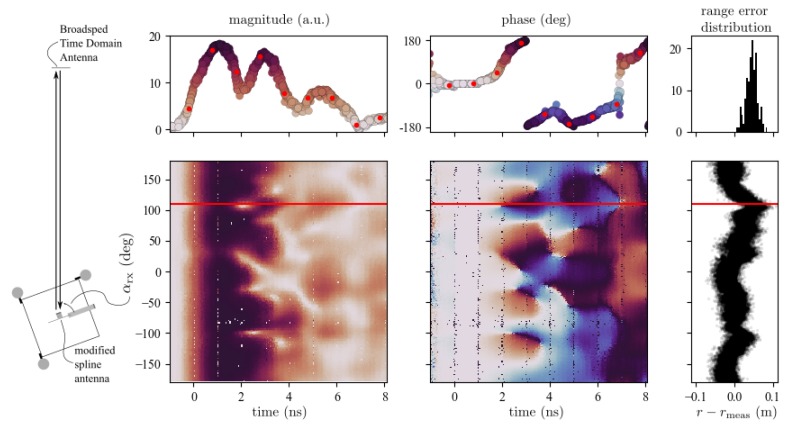
On the left, the experimental setup is shown with a modified spline antenna taking range and CIR envelope measurements for different AOAs to a Time Domain Broadspec antenna (pictures of both antennas are shown in Figure 2). The top plots show the magnitude and phase of over 100 accumulated and aligned CIR envelope measurements, and a histogram of the corresponding range error for an AOA of $\alpha_{\mathrm{rx}}=110{}^{\circ}$. A single CIR envelope measurement is highlighted with red dots. The bottom plots show these metrics for all different AOA using the same color scheme as in the top plots whose AOA is marked with a red line.

As the phase difference between the transmitter and receiver clock varies from one signal transmission to another due to clock imperfections, the CIR envelope samples obtained during each signal reception show different temporal locations and a different phase offset. To obtain a higher resolution of the CIR envelope, these accumulated CIR envelope measurements can be aligned by Decawave’s proprietary leading edge detection algorithm [31], as discussed in detail in [32]. This leading edge detection algorithm estimates the first path location within a CIR envelope with a resolution of 15.6 ps. Two plots in the top row of Figure 3 show such alignments for over 100 different CIR envelope measurements obtained with the modified spline antenna for an AOA of $\alpha_{\mathrm{rx}}=110{}^{\circ}$. One aligned CIR envelope measurement is highlighted with red dots and the time at the first path location is set to t=0. Note that in order to visualize the phase evolution, the phase offsets of the different aligned measurements are accounted for by setting the phase of the first sample after the estimated first path location to zero.

Figure 3 further shows how the phase and magnitude of the CIR envelope change for different AOA when employing the modified spline antenna. There are AOA ranges for which the magnitude and phase look completely different, but there are also AOA ranges for which they look similar, e.g., for CIR measurements obtained with AOAs at around −80° or at around −145°. Measurements for all the different tag antenna configurations can be found in Appendix B. As the timestamps used for the range measurement on the anchor and the tag are based on these CIR envelope measurements, changes in the CIR envelope can lead to errors in the timestamps, which in turn lead to errors in the measured TOF or range $r_{\mathrm{meas}}$. This is visible in the rightmost plot of Figure 3, where the differences of the measured range $r_{\mathrm{meas}}$ and the ground truth range *r*, provided by the motion capture system, for different AOAs and AODs are plotted (note that we use a two-way ranging protocol). Research to date has attempted to minimize these angle-dependent effects either by tailored antenna designs, evolved leading edge detection algorithms, or by calibration, as was discussed in Section 1.2. Instead of minimizing these effects, this paper suggests amplifying them such that useful information on the AOA can be retrieved.

Note that for notational simplicity, in the following we refer to the complex CIR response envelope simply as CIR.

## 3. Learning the CIR to AOA Mapping

### 3.1. Windowing

Looking at Equation (1), it is clear that in general the measured CIR is a result of different, multipath-dependent AOAs. Samples in the CIR far away from the estimated first path location are more likely to be the the result of a convolution with an antenna impulse response function corresponding to a multipath AOA. Therefore we only consider the CIR samples obtained within τns after the estimated first path sample. This τ is a tuning variable and defines an ellipsoid, with the transmitter and the receiver positions as foci points, denoted with $p_{\mathrm{tx}}$ and $p_{\mathrm{rx}}$, respectively. This ellipsoid is visualized in Figure 1 and is given by

(3) $$\begin{array}{c} ∥p_{\mathrm{tx}}-\left(\begin{array}{c} x\\ y\\ z \end{array}\right)∥+∥p_{\mathrm{rx}}-\left(\begin{array}{c} x\\ y\\ z \end{array}\right)∥=∥p_{\mathrm{rx}}-p_{\mathrm{tx}}∥+c\tau , \end{array}$$

with (x,y,z) the coordinates of the points lying on the surface of this ellipsoid, and with *c* the speed of light. Reflections occurring outside this ellipsoid only impact the measured CIR samples which are more than τns after the estimated first path sample. Ideally, free space can be assumed within the ellipsoid. However, apart from aerospace applications this is seldom the case as often the ground intersects with such an ellipsoid when operating close to ground. Nevertheless, multipath transmissions within the ellipsoid are likely to have a similar AOA as the direct path and ideally a model mapping the measured CIR to an AOA, as presented next, is robust to such multipath components.

### 3.2. CIR to AOA Mapping

The mapping of measured CIR to AOA is not one-to-one for general environments and antennas. On the one hand, different AOAs can result in a very similar antenna impulse response function as can be seen in Figure 3. This problem might be circumvented by an optimized antenna design or by optimally placing reflective surfaces around it. On the other hand, even if the antenna impulse response function was different for all AOA, differences in the environmental and transmitting antenna impulse response functions can again lead to the same measured CIR for different AOA. Hence, instead of learning a one-to one mapping of the measured CIR to the AOA, we propose to learn the probability that the measured CIR is the result of convolving $h_{\mathrm{tx}}*h_{\mathrm{env}}$ with $h_{\mathrm{rx}}\left(\alpha_{\mathrm{rx}}\right)$ to cope with this problem. This modeling is approached using a neural network trained on a large dataset of CIR measurements paired with the corresponding AOAs. Considering the environment’s influence on the measured CIR ($h_{\mathrm{env},n}$ in Equation (1)), this dataset ideally includes CIR measurements obtained in a similar environment as the application environment. Furthermore, training on multiple datasets obtained in different environments enables the neural network to better generalize to a new environment.

### 3.3. Network Structure

**Figure 4:**
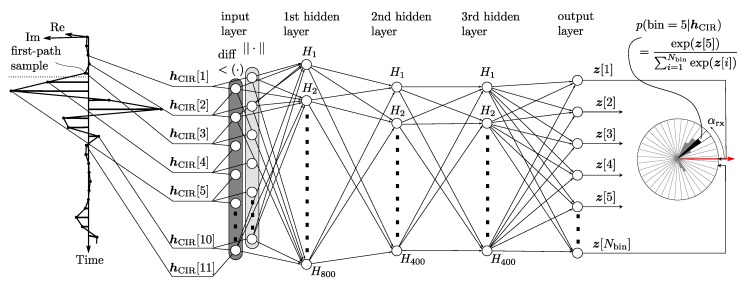
A standard neural network with three hidden layers is used to predict the AOA based on a window of the complex CIR envelope, starting two samples before the first path sample and ending eight samples after the first path sample. The magnitude and the phase differences of these eleven samples are fed to the input layer of the neural network. The outputs of the neural network are the unnormalized log probabilities of an AOA corresponding to a certain bin.

The conditional probability $p(\alpha_{\mathrm{rx}}|h_{\mathrm{CIR}}\left(t\right))$ is learned in a supervised learning framework in which a neural network is trained to minimize the cross-entropy between training data and the model distribution [33] (p. 173). The training data consists of of CIR and AOA pairs and is further described in Section 4. As previously discussed, the distribution $p(\alpha_{\mathrm{rx}}|h_{\mathrm{CIR}}\left(t\right))$ is expected to be multi-modal. Common approaches to learn such distributions using neural networks are mixture density networks [34] and histogram density estimations [35] (p. 120) in which the probability distribution is discretized. The latter was chosen due to ease of implementation and good stability during training. To this end, the AOA was discretized into $N_{\mathrm{bin}}=256$ bins and the neural network was trained to predict the unnormalized log probabilities that a signal is received with an AOA corresponding to a certain bin. This is visualized in Figure 4. The network consists of three fully connected layers of size 800, 400, 400 with rectified linear unit activation function ReLU [36]. The network’s input is a eleven sample window of the measured complex impulse response, starting two samples before and ending eight samples after the first path sample, and is denoted simply by $h_{\mathrm{CIR}}$ in the following to keep the notation concise. This corresponds to a τ=8 ns as given in Equation (3). The phase difference between consecutive samples in $h_{\mathrm{CIR}}$ is calculated and fed together with the magnitude of the first ten samples in $h_{\mathrm{CIR}}$ as a concatenated vector of 20 elements to the neural network. Feeding the phase differences instead of the measured phase sped up training as the neural network otherwise would have to implement a similar operation itself due to the previously discussed varying phase offset of the different CIR measurements.

Denoting with $z\in R^{N_{\mathrm{bin}}}$ the output of the neural network, i.e., the unnormalized log probabilities, the probability that the AOA of a signal falls into bin $i\in \{1,\;\ldots ,N_{\mathrm{bin}}\}$ is

(4) $$\begin{array}{c} p\left(\mathrm{bin}=i|h_{\mathrm{CIR}}\right)=\frac{\exp(z[i])}{\sum_{j=1}^{N_{\mathrm{bin}}}\exp\left(z\left[j\right]\right)}. \end{array}$$

### 3.4. Network Training

The network is implemented in Tensorflow [37] and trained using the ADAM optimizer [38]. We train the network to minimize the cross-entropy loss for all training data points $\left(\alpha_{\mathrm{rx}},h_{\mathrm{CIR}}\right)$, i.e.,

(5) $$\begin{array}{c} J\left(\alpha_{\mathrm{rx}},h_{\mathrm{CIR}}\right)=-\log p\left(\mathrm{bin}=\mathrm{bin}\left(\alpha_{\mathrm{rx}}\right)|h_{\mathrm{CIR}}\right), \end{array}$$

where $\mathrm{bin}(\alpha_{\mathrm{rx}})$ denotes the ground-truth bin of the training sample. Twenty percent of the training data is randomly chosen and retained as validation data. Dropout regularization (at a 10% rate) is employed after each hidden layer during training, which terminates when the loss on the validation data stops decreasing. The training batch size is 2000 and the training data is normalized to speed up training.

## 4. Acquiring the Datasets

**Figure 5:**
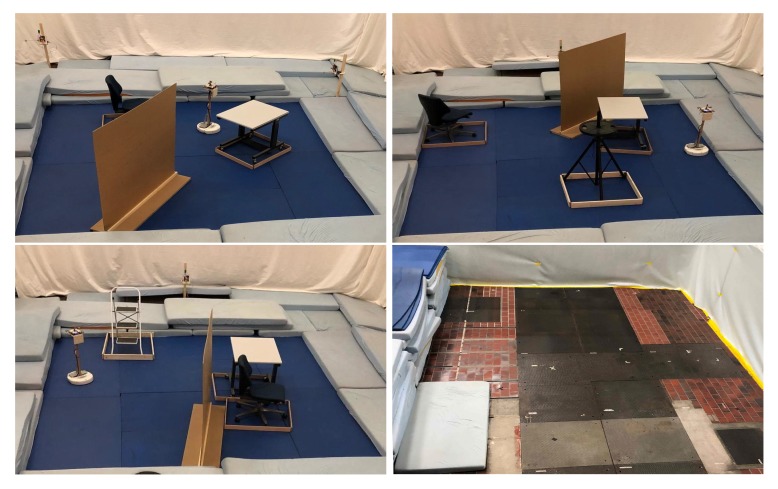
Three out of ten setups for data collection using the modified spline antenna are shown. In each setup the Roomba robot drove a different trajectory and the obstacles’ or the transmitters’ location was changed. The picture in the bottom right shows the floor made of ceramic tiles and metal plates.

**Figure A1:**
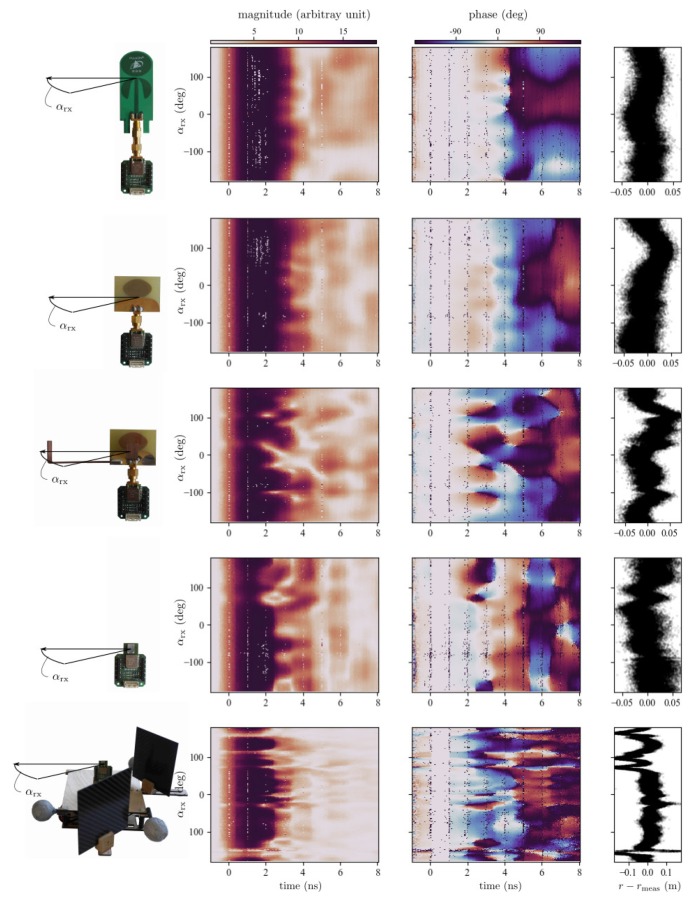
The complex CIR envelope for different receiver antenna configurations is shown for different AOA with the estimated first path location at t=0 ns. The transmission antenna is always a Broadspec Time Domain antenna.

In addition to the datasets used to qualitatively assess the AOA dependency of the measured CIR as discussed in Section 2, datasets using multiple anchors and changing environments were also collected. To this end, a Roomba robot equipped with a UWB tag drove around in a random fashion in an area of 4.2 m×4.3 m while recording the output of its wheel encoders with a frequency of 66 Hz and sequentially collecting CIR and range measurements to five anchor modules with a frequency of 200 Hz. Again the ranging protocol presented in [27] was employed and the CIR of the last anchor reply was recorded. The anchor modules were equipped with Broadspec Time Domain antennas because of their constant antenna impulse response function over different angles as visible in Figure A1. This limits the influence of the AOD on the measured CIR, which facilitates the CIR to AOA mapping problem. The antennas were placed 0.9 m above ground around the area the Roomba robot was driving in, such that range measurements ranging from 0.5 m to 9 m were obtained. Different obstacles, i.e., a chair, a table, a wooden wall, a ladder and a tripod, were placed in the area. For each collected dataset containing roughly 200’000 CIR and range measurements, the Roomba robot was traveling a different, random trajectory and either the locations of the anchor modules, or the locations of obstacles within the area were changed as shown in Figure 5. This figure also shows a picture of the floor, made of ceramic tiles and heavy metal plates, partially reflecting the UWB signals. Sport mattresses were placed on the floor to facilitate the Roomba robot’s locomotion. During the experiment, an overhead motion capture system recorded the ground truth position and orientation of the tag and anchor antennas with sub-centimeter and sub-degree accuracy. Synchronizing and processing all this data allows pairing of CIR measurements with the corresponding AOA to generate training and evaluation datapoints $\left(\alpha_{\mathrm{rx}},h_{\mathrm{CIR}}\right)$ for the previously presented neural network.

For ten such datasets, the UWB tag on the Roomba robot was equipped with the modified spline antenna; and for a further ten such datasets, the Partron dielectric chip antenna with mounted carbon plates in its vicinity was used, as shown in Figure 2. These datasets are made publicly available here [3].

## 5. Results

The neural network described in Section 3 was trained and evaluated with the previously described datasets, separately for the modified spline antenna and for the Partron dielectric chip antenna with carbon plates in its vicinity. From the ten datasets originating from different setups, nine datasets were used for training and the evaluation was made on the remaining dataset. This was done ten times each time leaving out a different dataset in training for the following evaluation. The results of this leave-one-out cross-validation are presented by means of the error in the maximum a-posteriori AOA estimate ${\hat{\alpha }}_{\mathrm{rx}}$. It is given as the bin center of the bin with the highest probability as predicted by the neural network, i.e.,

(6) $$\begin{array}{cc} \hat{\mathrm{bin}}:= & \underset{i\in 1,\;\ldots ,N_{\mathrm{bin}}}{arg max}p\left(\mathrm{bin}=i|h_{\mathrm{CIR}}\right) \end{array}$$

(7) $$\begin{array}{cc} {\hat{\alpha }}_{\mathrm{rx}}:= & 2\pi \frac{\hat{\mathrm{bin}}-1}{N_{\mathrm{bin}}}. \end{array}$$

As discussed in Section 2, the resolution of the measured CIR is relatively coarse at approximately 1 ns. However, as it sampled at slightly different locations is each time, a more accurate maximum a-posteriori estimate can be found by collecting 10 consecutive CIR measurements $h_{\mathrm{CIR}}\left(1\right),\;\ldots ,h_{\mathrm{CIR}}\left(10\right)$ from the same transmitter, and choosing the estimated AOA bin as

(8) $$\begin{array}{cc} \hat{\mathrm{bin}} & \underset{i\in 1,\;\ldots ,N_{\mathrm{bin}}}{arg max}\sum_{j=1}^{10}p\left(\mathrm{bin}=i|h_{\mathrm{CIR}}\left(j\right)\right). \end{array}$$

**Figure 6:**
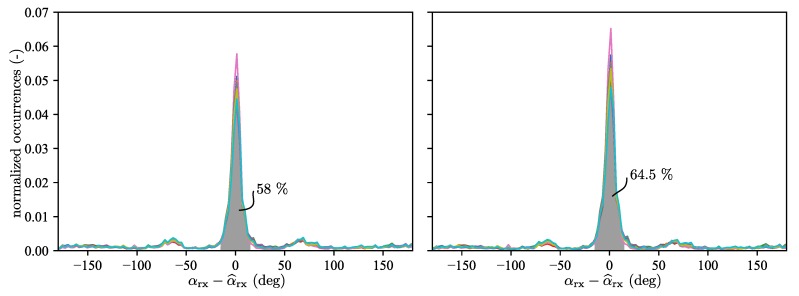
The error distribution of the maximum a-posteriori AOA estimate is shown for the ten different datasets using the modified spline antenna. On the left, the distribution is shown for when the maximum a-posteriori estimate is calculated using a single CIR. On average 58% of these estimates have an error of less than 15°. This value is increased to 64.5% when the maximum a-posteriori estimate is calculated using ten consecutive CIR measurements, which is shown on the right.

Figure 6 shows the error distribution for the modified spline antenna. Averaged over the datasets, 58% of the maximum a-posteriori estimates have an error of less than 15${}^{{}^{\circ}}$ when using only one CIR measurement. When using 10 consecutive CIR measurements, this ratio is increased to about 64.5%, which is still significantly lower than the ratio for the AOA estimation modules based on multiple antennas created by Ubisense [39] and Decawave [5]. Almost 100% of their AOA measurements are reported to be within a 15${}^{{}^{\circ}}$ error bound.

**Figure 7:**
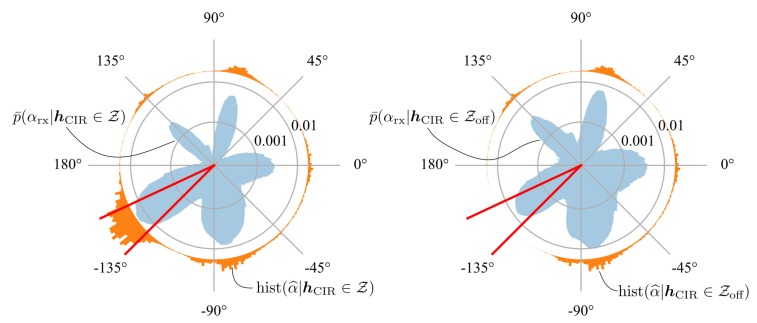
On the left, the averaged, multimodal probability distribution predicted by the neural network is shown in blue for CIR measurements obtained with AOA in the range of − 135° to − 155° (see Equations (9) and (10)). Also the histogram of the corresponding maximum a-posteriori AOA estimates is shown in orange. On the right, the averaged probability distribution is shown for CIR measurements obtained in the same range, but whose corresponding maximum a-posteriori AOA estimate also deviates by more than 30° (see Equations (11) and (12)).

However, the distribution predicted by the neural network is in general multimodal. Therefore, even if the maximum a-posteriori estimate might deviate by a large value, the probability of the bin corresponding to the ground-truth AOA $\mathrm{bin}(\alpha_{\mathrm{rx}})$ might still be high. This is visible in Figure 7 where the average predicted a-posteriori AOA probability distribution for CIR measurements belonging to the datapoints Z obtained with a ground truth AOA between −135 ${}^{{}^{\circ}}$ and −155 ${}^{{}^{\circ}}$ is shown, i.e.,

(9) $$\begin{array}{c} Z=\{\left(\alpha_{\mathrm{rx}},h_{\mathrm{CIR}}\right):-155{}^{{}^{\circ}}<\alpha_{\mathrm{rx}}<-135{}^{{}^{\circ}}\} \end{array}$$

(10) $$\begin{array}{c} \bar{p}\left(\alpha_{\mathrm{rx}}|h_{\mathrm{CIR}}\in Z\right)=\frac{1}{\left|Z\right|}\sum_{h_{\mathrm{CIR}}\in Z}p\left(\alpha_{\mathrm{rx}}|h_{\mathrm{CIR}}\right), \end{array}$$

where |Z| is used to denote the number of datapoints contained in set Z. On the right, the average predicted a-posteriori AOA probability distribution is shown for the subset of these datapoints $Z_{\mathrm{off}}\subset Z$, whose maximum a-posteriori AOA estimate deviates by more than 30${}^{{}^{\circ}}$, i.e.,

(11) $$\begin{array}{c} Z_{\mathrm{off}}=\{\left(\alpha_{\mathrm{rx}},h_{\mathrm{CIR}}\right):-155{}^{{}^{\circ}}<\alpha_{\mathrm{rx}}<-135{}^{{}^{\circ}}\;\mathrm{and}\;|{\hat{\alpha }}_{\mathrm{rx}}-\alpha_{\mathrm{rx}}\left|>30{}^{{}^{\circ}}\right\} \end{array}$$

(12) $$\begin{array}{c} \bar{p}\left(\alpha_{\mathrm{rx}}|h_{\mathrm{CIR}}\in Z_{\mathrm{off}}\right)=\frac{1}{|Z_{\mathrm{off}}|}\sum_{h_{\mathrm{CIR}}\in Z_{\mathrm{off}}}p\left(\alpha_{\mathrm{rx}}|h_{\mathrm{CIR}}\right). \end{array}$$

It is evident that most maximum a-posteriori AOA assigned to these CIR measurements are at around −80 ${}^{{}^{\circ}}$, even though the probability of the AOA corresponding to the actual AOA is still high. This can also explain the bump at around $-65{}^{{}^{\circ}}$ in maximum a-posteriori error distribution, as seen in Figure 6. Looking at Figure 3, it is evident that the antenna impulse response function for the AOAs from −135 ${}^{{}^{\circ}}$ to −155 ${}^{{}^{\circ}}$ seems to be similar to the one for the AOAs from −70 ${}^{{}^{\circ}}$ to −90 ${}^{{}^{\circ}}$. Therefore, the neural network has difficulty in mapping the measured CIR to the correct AOA. However, this uncertainty is also mirrored in the probability distribution given by the neural network.

Similar results were achieved with the Partron dielectric chip antenna with mounted carbon plates in its vicinity, as further discussed in Appendix C, which shows that material in the antenna’s vicinity influencing its radiation pattern also helps to estimate the AOA with the proposed method.

Although it may at first appear contrived, in the majority of applications the antenna’s radiation pattern is distorted, either because the antenna is integrated into the device case, or because the device case and the device electronics reflect and dampen electromagnetic waves in different manners depending on the device orientation. However, these unintentional angle-dependent radiation patterns lead in general to multimodal probability distributions, as is also the case for the tested modified spline antenna and the Partron dielectric chip antenna with carbon plates. In order to improve accuracy of the maximum a-posteriori AOA estimate, the antenna design or the placement of the reflective surfaces should be optimized, which was not done in this work. Nevertheless, there are applications where multimodal distributions pose less of a problem, e.g., when it is possible to fuse multiple AOA distributions from different transmitters or receivers, as is the case for UWB localization problems, which is discussed in the next section.

## 6. Application to a Self-Localization Problem

In this section, the previously described method to estimate the AOA based on CIR measurements is used to localize a robot.

### 6.1. Self-Localization Problem

**Figure 8:**
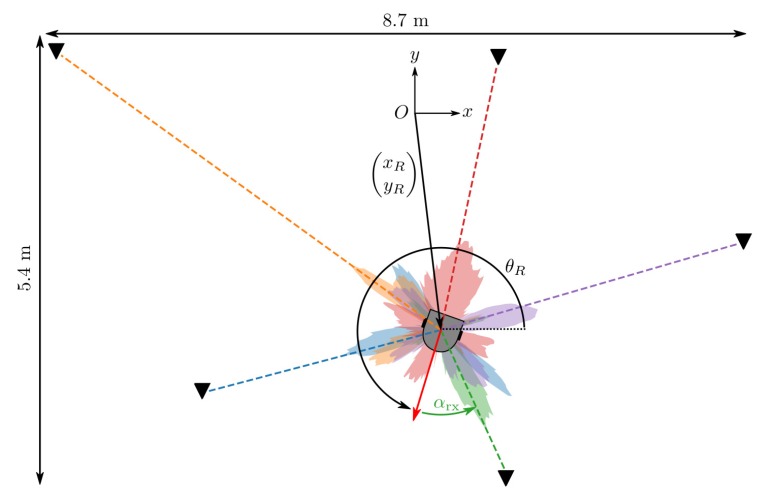
A robot can localize itself with respect to anchors having known locations by maximizing the AOA likelihood as given by the neural network. The predicted AOA a-posteriori probability distributions for each anchor are shown in different colors.

The datasets described in Section 4 were collected with the help of a mobile robot (Roomba) and consist of CIR, range and odometry measurements along with the ground truth measurements provided by a motion capture system. Given the trained neural network as outlined in Section 3, the CIR measurements with anchors at known locations provide sufficient information for the robot to estimate its state $x=(x_{R},y_{R},\theta_{R})$ in the inertial reference frame, where $x_{R}$ and $y_{R}$ are the robot’s Cartesian coordinates and $\theta_{R}$ is the angle describing its orientation (see Figure 8). The state estimate can be obtained via triangulation as visualized in Figure 8, e.g., by maximizing the measurement likelihood

(13) $$\begin{array}{c} x=arg max\prod_{h_{\mathrm{CIR}}\in Z}p\left(\alpha_{\mathrm{rx}}\left(x\right)|h_{\mathrm{CIR}}\right). \end{array}$$

In order to self-localize, the robot does not need to move as long as its position and the position of the anchors cannot be circumscribed with a circle [40]. This enables the robot to self-localize by only receiving the UWB signals from transmitters with a known location, which do not even need to be synchronized. It is clear that if time-of-flight measurements or time-of-flight-difference measurements with respect to the anchors are available, they significantly improve the performance of such a localization system and should therefore be fused with AOA measurements. The same applies to motion or process models, which should be used as well if available.

In the following, we will investigate the fusion of this information by means of a particle filter in order to assess the benefit of estimating the AOA with the proposed method in self-localization applications. Furthermore, such fusion approaches also allow the neural network to be trained without a motion capture system, as demonstrated in the following. A general comparison of time-of-flight, AOA, and received signal strength localization approaches is given in [1].

### 6.2. Particle Filter

Two discrete-time process models x(k+1)=q(x(k),u(k),η(k)) for the mobile robot are considered, where u(k) is the system’s input and $\eta \left(k\right)=(\eta_{x}\left(k\right),\eta_{y}\left(k\right),\eta_{\theta }\left(k\right))$ is the process noise at discrete time k=1,2,⋯ for a sampling period of 15 ms, which is equal to the period with which the Roomba robot’s wheel encoders can be recorded.

#### 6.2.1. Random Walk Process Model

In the random walk process model, the system input u is assumed to be zero and the state is assumed to evolve solely based on the process noise, i.e.,

(14) $$\begin{array}{cc} x_{R}\left(k+1\right) & =x_{R}\left(k\right)+\eta_{x}\left(k\right) \end{array}$$

(15) $$\begin{array}{cc} y_{R}\left(k+1\right) & =y_{R}\left(k\right)+\eta_{y}\left(k\right) \end{array}$$

(16) $$\begin{array}{cc} \theta_{R}\left(k+1\right) & =\theta_{R}\left(k\right)+\eta_{\theta }\left(k\right). \end{array}$$

The process noise is assumed to have a zero mean normal distribution, i.

(17) $$\begin{array}{c} \eta \left(k\right)∼N\left(\eta \left(k\right)|0,\Sigma \right)\;\mathrm{with}\;\Sigma =\left(\begin{array}{ccc} {\left(12mm\right)}^{2} & 0 & 0\\ 0 & {\left(12mm\right)}^{2} & 0\\ 0 & 0 & {\left(5.4{}^{{}^{\circ}}\right)}^{2} \end{array}\right). \end{array}$$

#### 6.2.2. Roomba Process Model

In the more accurate Roomba process model, the robots state x is pushed forward by the robot’s odometry recordings u=(Δp,Δθ), where Δp and Δθ are the measured distance travelled and the measured change in heading, respectively, during the sampling period. This process model is given as

(18) $$\begin{array}{cc} x_{R}\left(k+1\right) & =x_{R}\left(k\right)+\cos\left(\theta_{R}\left(k\right)\right)\Delta p\left(k\right)+\eta_{x}\left(k\right) \end{array}$$

(19) $$\begin{array}{cc} y_{R}\left(k+1\right) & =y_{R}\left(k\right)+\sin\left(\theta_{R}\left(k\right)\right)\Delta p\left(k\right)+\eta_{y}\left(k\right) \end{array}$$

(20) $$\begin{array}{cc} \theta_{R}\left(k+1\right) & =\theta_{R}\left(k\right)+\Delta \theta \left(k\right)+\eta_{\theta }\left(k\right). \end{array}$$

For this process model, the process noise covariance is lowered to

(21) $$\begin{array}{c} \Sigma =\left(\begin{array}{ccc} {\left(2mm\right)}^{2} & 0 & 0\\ 0 & {\left(2mm\right)}^{2} & 0\\ 0 & 0 & {\left(1.5{}^{{}^{\circ}}\right)}^{2} \end{array}\right). \end{array}$$

#### 6.2.3. Measurement Model

Measurement updates can be performed either with a-posteriori AOA probability distributions provided by the neural network, or with time-of-flight and the corresponding range measurements. These updates are further described in the following algorithm.

#### 6.2.4. Particle Filter Algorithm

How these process and measurement models can be integrated in a particle filter is briefly outlined in the following summary, and the reader is referred to [41] for a more in-depth introduction.

1. Initialization: The particle filter is initialized with $N_{\mathrm{PF}}=1000$ particles $x^{p},p\in \left\{1,2,\;\ldots ,N_{\mathrm{PF}}\right\}$ whose initial *x*, *y* coordinates and headings are drawn from the uniform distributions $U_{x_{R}}\left(0\;m,4.7\;m\right)$, $U_{y_{R}}\left(0\;m,4.8\;m\right)$ and $U_{\theta_{R}}\left(-\pi ,\pi \right)$.
2. Prediction step: At each iteration, the random walk (15) and (16) or the Roomba (19) and (20) process model is used to update each particle $x^{p}$ as
  (22) $$\begin{array}{c} x^{p}\left(k+1\right)=q\left(x^{p}\left(k\right),u\left(k\right),\eta^{p}\left(k\right)\right). \end{array}$$
3. Measurement update: When a UWB signal is received, the particle weights can be updated according to their likelihood given the current AOA a-posteriori probability distribution or the current range measurement. Using the AOA a-posteriori probability distribution, the particles weights are calculated as
  (23) $$\begin{array}{c} w^{p}\left(k\right)=p\left(\alpha_{\mathrm{rx}}^{p}\left(k\right)|h_{\mathrm{CIR}}\left(k\right)\right), \end{array}$$
  where the expected AOA $\alpha_{\mathrm{rx}}^{p}$ of each particle p is
  (24) $$\begin{array}{c} \alpha_{\mathrm{rx}}^{p}\left(k\right)=\mathrm{atan}2\left(y_{A}-y_{R}^{p}\left(k\right),x_{A}-x_{R}^{p}\left(k\right)\right)-\theta_{R}^{p}\left(k\right), \end{array}$$
  wherein $x_{A}$ and $y_{A}$ are the *x* and *y* coordinates of the anchor modules from which a signal is received. If the range measurement is used, the particle weights are calculated as
  (25) $$\begin{array}{c} w^{p}\left(k\right)=p\left(r^{p}\left(k\right)|r_{\mathrm{meas}}\left(k\right)\right)∼N\left(r^{p}\left(k\right)|r_{\mathrm{meas}}\left(k\right),\sigma_{r,\mathrm{meas}}^{2}\right), \end{array}$$
  with $r_{\mathrm{meas}}$ the measured range with a variance of $\sigma_{r,\mathrm{meas}}^{2}$, and where the expected range $r^{p}$ of each particle *p* is calculated as
  (26) $$\begin{array}{c} r^{p}\left(k\right)=∥\left(\begin{array}{c} x_{R}\left(k\right)\\ y_{R}\left(k\right) \end{array}\right)-\left(\begin{array}{c} x_{A}\\ y_{A} \end{array}\right)∥. \end{array}$$
  After the particle weights have been calculated, the particles are resampled to get $N_{\mathrm{PF}}$ posterior particles, all with equal weights.

### 6.3. Training with Particle Filter AOA Data

So far, the training data for the neural network was obtained by means of a motion capture system, i.e., the AOA corresponding to a measured CIR was calculated based on motion capture data. With these training data, the neural network described in Section 3 was trained. However, the AOA corresponding to a measured CIR can also be obtained by other means; namely, based on the estimated state by the particle filter fusing odometry and range measurements as outlined above. The data obtained might be of lower quality, i.e., the AOA assigned to a measured CIR might deviate if the estimated state also deviates. Nevertheless, as long as the data is unbiased, the neural network can be successfully trained with it.

To investigate this, ten new training datasets were generated for the modified spline antenna where the AOA was not provided by the motion capture system, but by the state estimate of the particle filter employing the Roomba process model to fuse odometry and range measurements. The particle filter’s position, ${\hat{p}}_{R}=\left({\hat{x}}_{R},{\hat{y}}_{R}\right)$, and orientation, ${\hat{\theta }}_{R}$, estimates are defined to be the particles’ average position and orientation, respectively. Using these estimates, the AOA corresponding to a CIR measurement obtained at time *k* was calculated as

(27) $$\begin{array}{c} \alpha_{\mathrm{rx}}=\mathrm{atan}2\left(y_{A}-{\hat{y}}_{R}\left(k\right),x_{A}-{\hat{x}}_{R}\left(k\right)\right)-{\hat{\theta }}_{R}\left(k\right). \end{array}$$

These ten new datasets were again used to train the neural network in a leave-one-out cross validation fashion. The error distribution of the maximum a-posteriori AOA estimate does not differ significantly from the distribution obtained with neural networks trained on motion capture training data, but the value of the maximum probability density is generally smaller. This can be explained by the additional noise now included in the training data, which acts as a regularizer and leads to more conservative, i.e., more uniform, a-posteriori AOA probability distributions predicted by the neural network.

### 6.4. Results

**Figure 9:**
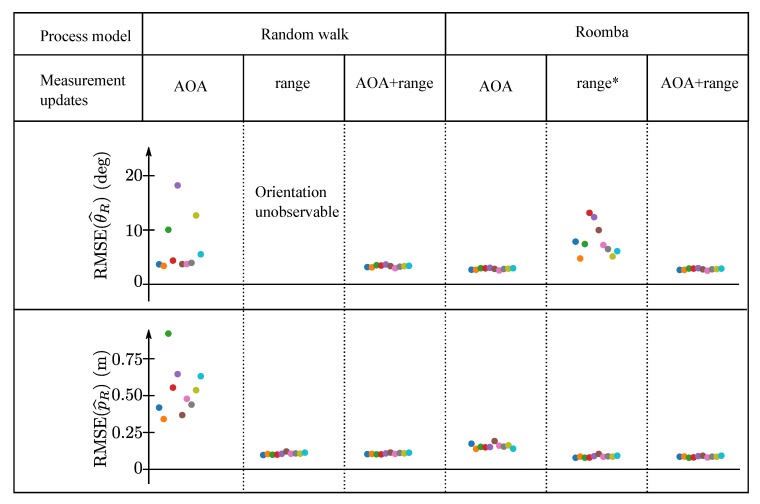
This figure compares the performance of particle filters having different process models and different measurement updates. The root mean square error (RMSE) obtained for the ten different leave-one-out cross-validation evaluations are shown in different colors. The neural networks employed by the particle filters using AOA measurement updates were trained with datasets in which the AOA corresponding to a CIR measurement was provided by a particle filter employing the Roomba process model and range measurement updates (see the column marked with * for its performance).

These newly trained neural networks were employed by the particle filter whose performance was evaluated for the two different process models, and the different measurement updates. A leave-one-out cross validation is again applied for the evaluation, in which the dataset not used for the neural network training was used for the evaluation. Figure 9 shows the root mean square error (RMSE) in the heading, $\mathrm{RMSE}({\hat{\theta }}_{R})$, and position, $\mathrm{RMSE}({\hat{p}}_{R})$, estimates of the particle filter for the different configurations. The RMSE for each dataset is shown in a different color.

In case of the random walk process model, it is apparent that the orientation and position of the Roomba robot can be estimated solely based on AOA measurements in all ten datasets. When employing range measurement updates instead of AOA measurements, the error in the position estimate is significantly smaller. However, it is no longer possible to observe the orientation of the robot. The best performance is achieved when range and AOA measurements are combined. Note that two-way communication between the mobile robot and the anchors or clock synchronization is necessary in order to obtain range measurements, whereas the AOA estimation method presented only needs one-way communication and no clock synchronization.

In the case of the particle filters employing the Roomba process model, the orientation of the Roomba robot is also observable without AOA measurements. This allowed the neural network to be trained without motion capture data as described in Section 6.3. Note that even though the RMSE in the heading of this particle filter employing the Roomba process model and range measurement updates was between 4${}^{{}^{\circ}}$ and 13${}^{{}^{\circ}}$ depending on the dataset (see column marked with * in Figure 9), neural networks trained with these data and integrated in a particle filters lead to RMSE in the heading of below 4${}^{{}^{\circ}}$ as also visible in Figure 9. Also for the Roomba process model, the best performance is achieved when range and AOA measurement updates are used.

The results of particle filters employing neural networks trained with motion capture data are included for completeness in Appendix D.

## 7. Conclusions

This paper discusses a technique to estimate the AOA of a UWB signal based on CIR measurements. We identify that the antenna’s impulse response function is AOA dependent, and that objects in the antenna’s local environment create angle-dependent reflections that further affect the measured CIR. We use a neural network to learn the mapping between the CIR measurement and the AOA, and show that the UWB signal’s AOA can be estimated at no additional hardware cost, using just a single antenna, unlike conventional AOA estimation techniques. By combining AOA estimates to multiple fixed-location UWB anchors, we experimentally demonstrate the localization of a mobile robot, based only on AOA estimates obtained from CIR measurements (see Supplementary Video), and in combination with range measurements. Given that in most real-world UWB applications the antennas’ impulse response functions are AOA dependent due to their integration into a device, we regard the AOA estimation method presented in this paper as a low-cost and software-only augmentation for any existing UWB TOF-based localization system, with which AOA-CIR training data can be collected on the go by data fusion approaches.

### Outlook

The new AOA estimation principle presented in this paper should be investigated further to assess its full potential and its limitations. We regard the following topics as interesting to investigate in the future:

**Hardware optimizations:** In this paper, we changed the antenna’s impulse response function in a straightforward manner by modifying the antenna, or by placing carbon plates in its vicinity. Instead, better performance could be achieved if the antenna design or the placement of the reflective surfaces were optimized for the application at hand considering the selected carrier frequency and power settings. Such optimizations, performed via electromagnetic simulation software, could be aimed at rendering the a-posteriori AOA probability distribution unimodal, and at making the method more robust to non-line-of-sight conditions. Furthermore, as the CIR estimates provided by the DW1000 chips is also dependent on the clock speed of the receiver and transmitter, more stable clocks and shorter sampling periods could further help to improve the accuracy of the AOA estimation method, although this would again lead to increased hardware costs.

**AOA of multi-path components:** As shown in [42,43], if the locations of reflective surfaces in the environment are known, the timing of multi-path components can be used to localize a receiver. In this paper we trim multi-path components, and focus only on the first peak. However, these multi-path components are also affected by the receiving antenna’s AOA-dependent transfer function as shown in [44], and it should be possible to also compute their AOAs using the techniques discussed in this paper.

**Learning:** The neural network applied to learn the mapping between the measured CIR and the AOA worked without significant tuning, however resulted in a binned probability distribution. It would be interesting to investigate whether accuracy could be improved using mixture-density networks, resulting in a continuous probability distribution, or using neural networks with complex weights. In the latter case, the complex envelope of the CIR could be fed directly to the neural network instead of feeding it via its magnitude and phase. This, together with a tailored network architecture could further improve the performance of this method.

**AOD and AOA estimation:** The CIR is affected by both the receiver’s and transmitter’s antenna, and by obstacles in their local environments. In order to minimize the influence of the transmitting antenna on the measured CIR and thus simplify measurement of the AOA, we outfitted transmitters with antennas having a very uniform transfer function (Time Domain Broadspec antenna). However, if both receiver and transmitter were equipped with antennas having strongly angle dependent impulse response functions, it should be possible to estimate both AOA and AOD from a single CIR measurement. In combination with a TOF method to estimate range, this would enable estimation of the full relative pose of the receiver with respect to the transmitter and thus localization of the receiver using just a single anchor.

## Figures and Tables

**Figure A2:**
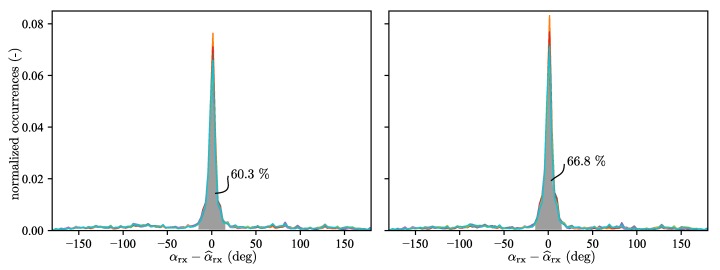
The error distribution of the maximum a-posteriori AOA estimate is shown for the ten different datasets using the Partron dielectric chip antenna with mounted carbon plates. On the left, the distribution is shown when the maximum a-posteriori estimate is calculated using a single CIR. On average 60.3% of the estimates have an error of less than 15${}^{{}^{\circ}}$. This value is increased to 66.8% when the maximum a-posteriori estimate is calculated using ten consecutive CIR measurements as shown on the right.

**Figure A3:**
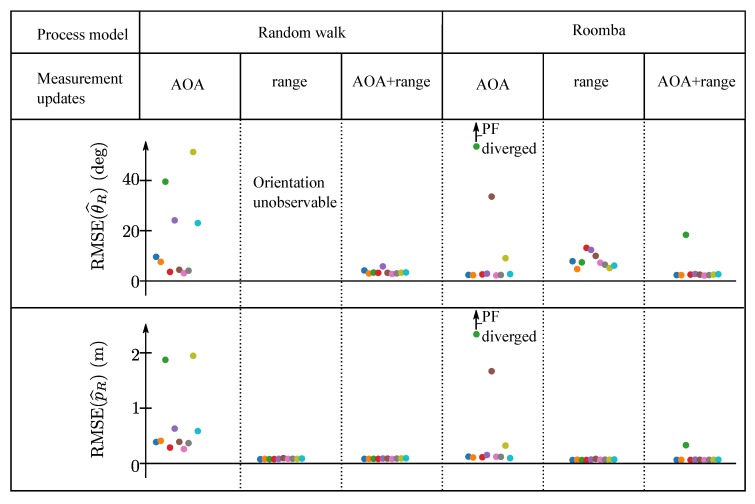
This figure compares the performance of particle filters having different process models and different measurement updates. The RMSE obtained for the ten different leave-one-out cross-validation evaluations are shown in different colors. The neural networks employed by the particle filters using AOA measurement updates were trained with datasets in which the AOA corresponding to a CIR measurement was provided by a motion capture system.

## Supplementary Materials

The following are available online at https://www.mdpi.com/1424-8220/19/20/4466/s1, Video S1: Ultra-wideband angle of arrival estimation based on angle-dependent antenna transfer function.

## Abbreviations

The following abbreviations are used in this manuscript:

AOA	Angle of Arrival
AOD	Angle of Departure
CIR	Channel Impulse Response
RMSE	Root-Mean-Square Error
TOF	Time of Flight
TOFD	Time of Flight Differences
UWB	Ultra-wide Band

## Appendix A. DW1000 Configuration

The configuration of the DW1000 chips is given in Table A1 and is used for all experiments. Note that mainly the center frequency and the output power were found to have a large impact on the measured CIR, even though no thorough experiments were conducted with different settings.

**Table A1:** The settings of the DWM1000 modules used to acquire the CIRs and range measurements.

Channel Number	4 (Carrier Frequency 3993.6 MHz, Bandwith 900 MHz)
Pulse Repetition Frequency	16 MHz
Data Rate	6.8 Mbps
Preamble Length	128 Symbols
Preamble Accumulation Size	8
Preamble Code	7
Transmit Power Control	19 dB Gain

## Appendix B. CIR Measurements for Different Antenna Configurations

Figure A1 compares the CIR measurements obtained with different receiver antenna configurations. Even though these measurements were not made in an anechoic chamber, it is qualitatively visible how the antenna impulse response functions of the different receiver antenna configurations impact the measured CIR, as the environment and the transmitter antenna were left unchanged for all collected measurements. Apart from antenna impulse response functions of the Broadspec Time Domain and the spline antennas, all other antenna impulse response functions seem to be clearly AOA-dependent.

## Appendix C. Results Obtained with Partron Dielectric Chip Antenna with Carbon Plates

The ten datasets recorded with the Partron dielectric chip antenna with mounted carbon plates are similar, but not identical to the ten datasets recorded with the modified spline antenna, as the anchor and obstacles locations were changed and as the Roomba robot for each experiment traveled a different path. Nevertheless, assuming that these differences are leveled out, the receiver configuration with the Partron dielectric chip antenna and the carbon plates seems to slightly decrease the error in the maximum a-posteriori AOA estimate as shown in Figure A2. Compared to the 58% and 64.5%, now 60.3% and 66.8% of the estimates have an error of less than 15${}^{{}^{\circ}}$, when using a single CIR or when using ten consecutive CIR measurements, respectively. As neither the placement of the carbon plates, nor the antenna design were optimized, no concluding statement can be made on which of two antenna radiation pattern influences can be better exploited by the AOA estimation method. However, carbon plates in the antenna’s vicinity seem to have a large impact on the error in the obtained range measurements, as visible in Figure A1, which should be compensated for when using range measurements.

## Appendix D. Results Obtained with Particle Filter Employing Neural Networks Trained with Motion Capture Data

The particle filters employing neural networks trained with motion capture data generally performed worse than the particle filters employing neural networks trained with fused data as outlined in Section 6.3. This is visible when comparing the RMSE in the heading and position estimates of these particle filters shown in Figure A3 with the ones shown in Figure 9 (note the different scales of the RMSE-axis when comparing the figures). When employing the Roomba process model and only AOA measurement updates, the particle filter even diverged for one dataset. As mentioned in Section 6.3, the neural network trained with fused data had a similar error distribution of their maximum a-posteriori AOA estimates, but generally predicted more conservative a-posteriori AOA-probability distributions. This could explain why the particle filters employing these models were less likely to diverge due to a erroneous maximum a-posteriori AOA estimate. Potentially, particle roughening, a higher process noise covariance or adding noise to the neural network training data could help to prevent the particle filter from diverging when employing the neural network trained with motion capture data.
